# A Decellularized Uterine Endometrial Scaffold Enhances Regeneration of the Endometrium in Rats

**DOI:** 10.3390/ijms24087605

**Published:** 2023-04-20

**Authors:** Yushi Yoshimasa, Tomoka Takao, Satomi Katakura, Shoko Tomisato, Hirotaka Masuda, Mamoru Tanaka, Tetsuo Maruyama

**Affiliations:** 1 Department of Obstetrics and Gynecology, Keio University School of Medicine, 35 Shinanomachi, Shinjuku-ku, Tokyo 160-8582, Japan; 2 Department of Regenerative Science, Graduate School of Medicine, Dentistry and Pharmaceutical Sciences, Okayama University, 2-5-1 Shikatacho, Kita-ku, Okayama-shi, Okayama 700-8558, Japan; 3 Department of Obstetrics and Gynecology, Tokyo Saiseikai Central Hospital, 1-4-17 Mita, Minato-ku, Tokyo 108-0073, Japan; 4 HM Ladies Clinic Ginza, 3-4-16 Ginza, Chuo-ku, Tokyo 104-0061, Japan

**Keywords:** ECM (extracellular matrix), scaffold, endometrium, regenerative medicine

## Abstract

Partial or whole regeneration of the uterine endometrium using extracellular matrix (ECM)-based scaffolds is a therapeutic strategy for uterine infertility due to functional and/or structural endometrial defects. Here, we examined whether the entire endometrium can be regenerated circumferentially using an acellular ECM scaffold (decellularized endometrial scaffold, DES) prepared from rat endometrium. We placed a silicone tube alone to prevent adhesions or a DES loaded with a silicone tube into a recipient uterus in which the endometrium had been surgically removed circumferentially. Histological and immunofluorescent analyses of the uteri one month after tube placement revealed more abundant regenerated endometrial stroma in the uterine horns treated with tube-loaded DES compared to those treated with a tube alone. Luminal and glandular epithelia, however, were not fully recapitulated. These results suggest that DES can enhance the regeneration of endometrial stroma but additional intervention(s) are needed to induce epithelization. Furthermore, the prevention of adhesions alone allowed the endometrial stroma to regenerate circumferentially even without a DES, but to a lesser degree than that with a DES. The use of a DES together with the prevention of adhesions may be beneficial for efficient endometrial regeneration in the uterus that is largely deficient of endometrium.

## 1. Introduction

Uterine infertility is one of the most common types of infertility in women, and reportedly occurs in 3–5% of the total population [1]. Uterine infertility can be classified into congenital and acquired infertility [2]. Congenital cases include Mayer-Rokitansky-Küster-Hauser syndrome, uterine hypoplasia and uterine malformation. Acquired infertility includes Asherman’s syndrome (intrauterine adhesions) and total or partial hysterectomy due to benign tumor, malignant tumor, postpartum bleeding, and transgender. For structural defects of the whole uterus, whether congenital or acquired, surrogacy and uterine transplantation are the most likely treatment options. However, these options are accompanied by numerous ethical, safety, and technical issues [3]. For instance, a lack of donors, high cost, and the need for long-term immunosuppressive drugs are serious obstacles for uterine transplantation [2,3,4].

Considering these obstacles, uterine regeneration technology is highly desirable to treat infertility due to whole uterus defects [2]. In general, a sufficient amount of a cell/tissue-supporting material termed a scaffold is necessary to efficiently achieve regeneration and reconstruction of organs and tissues of interest [3,5,6,7,8,9]. Indeed, we and several groups have prepared acellular extracellular matrices from the uteri of various animals, including mice, rats, pigs, and rabbits, through the removal of cells inside (decellularization) the uterus and have succeeded in the partial regeneration of the uterus using decellularized uterine scaffolds in various corresponding animals [3,9,10,11,12,13,14,15,16,17]. There have been, however, no reports of artificial regeneration of the entire uterus that mainly consists of endometrium and myometrium.

Importantly, the endometrium functions as the front line for direct interactions with embryos and successful support and maintenance of the fetoplacental unit. Therefore, structural and functional disturbances of the endometrium such as Asherman’s syndrome, recurrent implantation failure, and thin endometrium result in infertility, pregnancy loss, and other perinatal complications even in the presence of the healthy myometrium [18]. Thus, regeneration of healthy endometrium is an important therapeutic strategy for functional and structural defects of the endometrium [2,19,20]. However, regeneration of the entire endometrium has not yet been reported.

In this study, we aimed to regenerate the entire endometrium in rats using a decellularized endometrial scaffold (DES). We found that the prevention of adhesions together with the use of DES may more efficiently regenerate the entire endometrium than the use of DES alone.

## 2. Results

### 2.1. Characteristics of the Peeled-Off Endometrium

**Figure 1:**
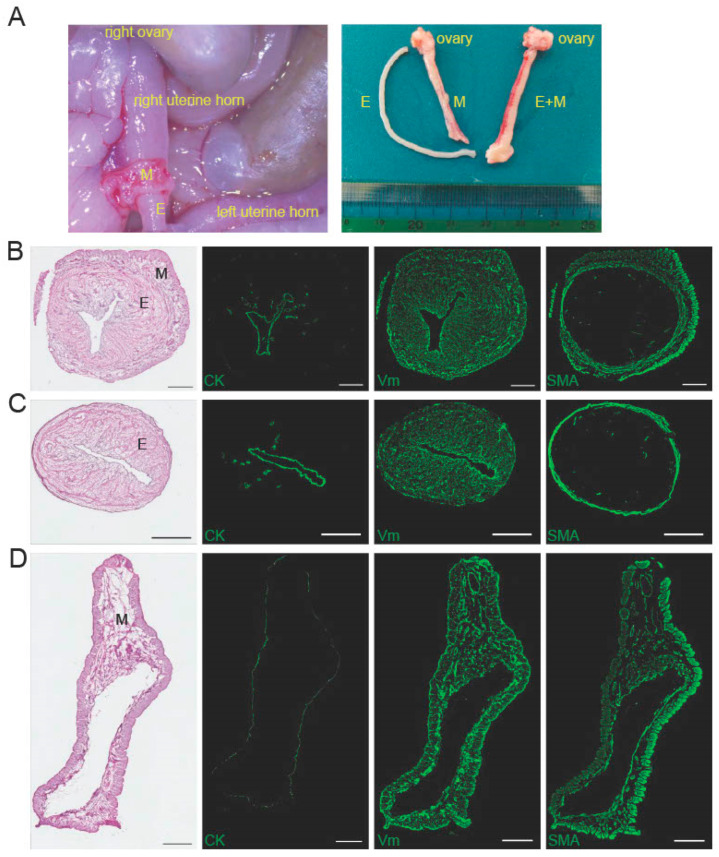
Isolation and characterization of the entire rat endometrium. (**A**) The entire endometrium (E) was separated and peeled off from the outer layer of the uterus containing myometrium (M) (left panel). A representative macroscopic image of the peeled-off E, separated M, and the whole uterine horn (E + M) is shown in the panel on the right. (**B**) As a positive control, the whole uterus was cross-sectioned and used for histology with hematoxylin and eosin (H&E) staining (left-most panel) and immunofluorescence staining using antibodies against cytokeratin (CK, green), vimentin (Vm, green), and smooth muscle actin (SMA, green) as indicated. (**C**) The peeled endometrium and (**D**) remaining outer layer of the uterus was cross-sectioned and analyzed as described in (**B**). Scale bars, 500 µm.

We peeled off the entire endometrium in a tubular form from rat uteri as shown in Figure 1A (left panel) and Video S1. The outer layer of the uterus containing myometrium was separated from the endometrium. Both the outer layer and the peeled endometrium retained an intact tubular shape (Figure 1A, right panel). Cross-sectional images of the whole uterus, the peeled endometrium, and the remaining outer layer of the uterus are shown in Figure 1B–D. Histology revealed that the tubular structure was preserved in both the peeled-off endometrium and the remainder of the outer layer of the uterus (Figure 1B–D left-most panels). Immunofluorescence staining showed that the whole uterus had luminal and glandular epithelial structures positive for cytokeratin (CK). The surrounding stroma was positive for vimentin (Vm), but negative for smooth muscle actin (SMA), and surrounding circumferential muscle layer was positive for both Vm and SMA (Figure 1B). Likewise, the peeled tubular endometrium possessed intact CK-positive epithelial structures and the entire surrounding Vm-positive and SMA-negative endometrial stroma, but the serosa was absent, and the circumferential muscle layer was thin relative to the whole uterus (Figure 1C). Conversely, the rest of the outer layer of the uterus had a serosa and Vm- and SMA-positive muscle layer together with tubular structures (Figure 1D).

### 2.2. Preparation and Characterization of a Decellularized Endometrial Scaffold (DES)

**Figure 2:**
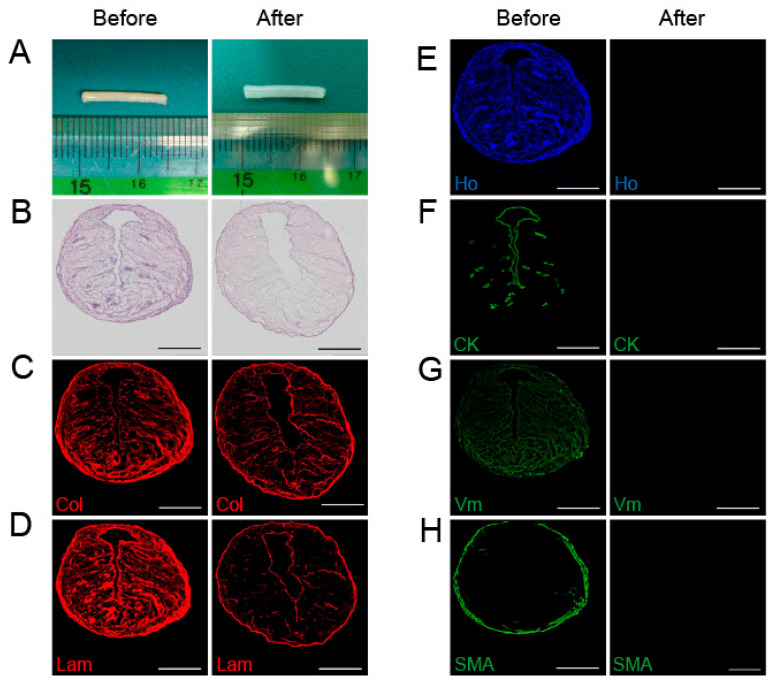
Preparation and characterization of decellularized rat endometrial scaffolds (DES). (**A**) The excised cylindrical endometrium was cut into 1.5 cm long pieces. (**B**–**H**) Endometrial pieces before (left panels) and after (right panels) decellularization were cross-sectioned and subjected to H&E staining (**B**) and immunofluorescence staining using antibodies against collagen type I (Col) (**C**), laminin (Lam) (**D**), Hoechst (Ho) (**E**), CK (**F**), Vm (**G**), and SMA (**H**). Scale bars, 500 µm.

The peeled-off tubular endometrium was cut into 1.5 cm long pieces and decellularized in SDS with shaking. Figure 2A shows macroscopic images of an endometrial piece before and after decellularization. H&E staining revealed that the decellularized endometrial scaffold (DES) maintained a tubular structure but had no cellular components (Figure 2B). The DES was positive for important extracellular matrix proteins, including collagen type I (Col) and laminin (Lam) (Figure 2C,D) that support tissue architecture, but was negative for Hoechst nuclear staining dye (Figure 2E) and cytoplasmic proteins such as CK, Vm, and SMA (Figure 2F–H).

### 2.3. Transplantation of DES with and without a Silicone Tube

**Figure 3:**
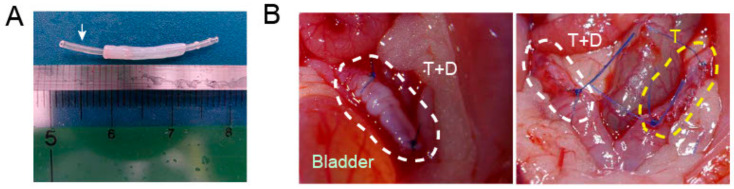
Placement of a silicone tube with or without a DES into endometrium-defective uterus in rats. (**A**) A silicon tube (white arrow) inserted into the lumen of cylindrical DES. (**B**) A tube alone (T) or tube-loaded DES (T+D) was placed into the endometrium-defective site of each uterine horn (circled by white and yellow dotted lines, respectively). The muscle layer and serosa were continuously sutured.

To investigate the endometrial regeneration potentials of DES in vivo, a silicone tube was inserted into the lumen of a cylindrical DES (Figure 3A) and the tube-loaded DES (T+D) was placed onto the endometrium-deficient site of a unilateral uterine horn in recipient rats (Figure 3B). As the control, a silicone tube alone (T) was placed similarly in the contralateral uterine horn (Figure 3B). The silicone tubes were used based on our earlier findings, showing that in the absence of tube placement, uterine occlusions and hydrometra related to intrauterine adhesions occurred in almost all animals that underwent in vivo regeneration experiments irrespective of the presence of a DES.

### 2.4. Histological and Immunofluorescent Analyses

**Figure 4:**
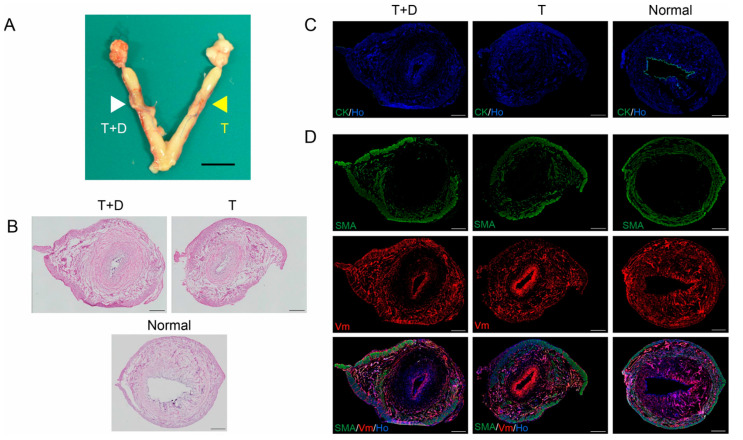
In vivo regeneration of the endometrium, following the placement of a silicone tube alone or a tube-loaded DES (T+D) in rats. (**A**) Representative image of the uterus excised from the rat treated for one month, showing that each horn treated with T+D (white arrowhead) or T (yellow arrowhead) was grossly normal. (**B**–**D**) Uterine horn that underwent no treatment (Normal) or the indicated treatment was cross-sectioned and subjected to H&E staining (**B**) or immunofluorescence staining using antibodies against CK (**C**), SMA ((**D**), upper panels), and Vm ((**D**), middle panels). Nuclei were stained with Hoechst (Ho). Scale bars, 500 µm.

We placed T or T+D individually into each uterine horn of six rats and excised the treated uteri one month later. Upon removal, each horn treated with T+D (white arrowhead) or T (yellow arrowhead) was grossly normal (Figure 4A).

Among the 12 treated uterine horns, one horn treated with T+D had an adhesion of the intrauterine cavity and exhibited resultant uterine distention due to hydrometra that precluded subsequent histological and immunofluorescence analyses. Data for this rat were excluded from further analyses and a total of 10 paired uteri were analyzed by H&E and immunofluorescence staining (Figure 4B–D).

H&E staining showed the preservation of luminal-like structures in both T+D and T groups, but the area of the stroma surrounding the luminal structure appeared to be smaller in the T group relative to the T+D group (Figure 4B). Immunofluorescence studies revealed that CK was absent in both T and T+D groups (Figure 4C) despite the presence of a luminal-like structure (Figure 4C). Interestingly, not all but many of the immunofluorescent samples exhibited intense Vm signals in the luminal-like structure in both T+D and T groups as shown in Figure 4D (middle and bottom panels). Silicone products are known to provoke fibrosis accompanied with Vm expression [21,22]. It is possible that the up-regulation of Vm expression may be attributable to the silicone tube-induced fibrosis. In contrast, co-immunostaining showed a SMA- and Vm-positive myometrial layer and the presence of a stroma that was positive for Vm but negative for SMA (Figure 4D). The stroma area and cell number were larger in the uterine horn treated with T+D as compared to the horn treated with T (Figure 4D).

**Figure 5:**
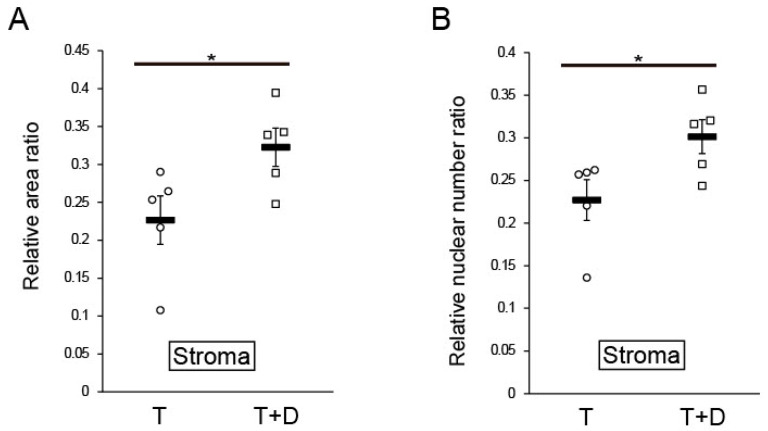
Quantitative analysis of regeneration potentials of silicone tube only (T) and tube-loaded DES (T+D). Based on the assumption that regeneration potential positively correlates with the area and cell (nuclei) number of the newly generated stroma, the total uterus/stroma ratio for these two parameters was calculated (**A**,**B**), as described in the text. The mean values of the relative area and nuclear number ratios obtained from the untreated normal tissue were 0.513 and 0.444, respectively. Values are expressed as mean ± SEM together and shown in dot plots of individual values obtained from the five independent experiments. * *p* < 0.05.

In a quantitative and statistical analysis, we next calculated ratios of the area and cell number in the stroma and myometrium to the total area (Figure 5). The ratio of the stromal region area to the total area was significantly higher for the T+D group than the T group (0.32 ± 0.025 vs. 0.23 ± 0.032, *p* < 0.05 by Student’s *t*-test) (Figure 5A). Likewise, the ratio of the number of nuclei in the stroma to that in the total uterus was also significantly higher for the T+D group than the T group (0.30 ± 0.020 vs. 0.23 ± 0.024, *p* < 0.05 by Student’s *t*-test) (Figure 5B). Importantly, the absolute area and nuclear number for the myometrium did not significantly differ between T and T+D (4.43 ± 0.11 mm^2^ vs. 4.74 ± 0.22 mm^2^, 12,914 ± 1260 cells vs. 13,787 ± 1983 cells, respectively), indicating that the area and nuclear number of endometrial stroma determined the ratio of the area and nuclear number of the stroma and myometrium.

In this study, the stage of the estrous cycle of each rat was not considered and, therefore, might be different among the rats. To eliminate a possible influence from intra-group variance in the estrous cycle, we calculated and compared the T+D:T ratio of the two parameters of each rat. The paired analysis revealed that the T+D:T ratio of the relative stromal area ratio was 1.35 (1.13–2.32):1 (median (25–75%), *p* < 0.01, Mann–Whitney test) and that the T+D:T ratio of the relative nuclear number ratio was 1.24 (1.15–1.86):1 (median (25–75%), *p* < 0.01, Mann–Whitney test). These results suggest DES enhanced the regeneration of endometrial stroma irrespective of the estrous cycle.

## 3. Discussion

The rodent uterus is connected to the dorsal body wall through heavy broad ligaments termed mesometria that contain blood and lymphatic vessels as well as abundant nerves [23]. Thus, uterine blood circulation is supplied through blood vessels bundled on the mesometrial side of the uterus [24]. Most studies to date, including ours, showed that natural and/or synthetic scaffolds could repair partial defects of the uterus, including in the myometrium and endometrium on the antimesometrial side [2,3,9,11,25,26,27,28,29,30,31,32]. Since the endometrium at the mesometrial side directly contacts and interacts with implanted embryo(s) [33], a rodent model having defects in the mesometrial endometrium is needed to examine the potential of natural and/or synthetic scaffolds to give rise to functionally receptive mesometrial endometrium. However, no studies have demonstrated the potentials of any scaffolds because the resection of the uterus, including the endometrium, at the mesometrial side typically causes excessive bleeding. In this study, we successfully removed the entire (i.e., both mesometrial and antimesometrial) endometrium circumferentially, and demonstrated for the first time that a DES had the potential to give rise to, at least in part, mesometrial endometrium stroma.

Many previous studies have developed various models of Asherman’s syndrome in which physical scraping methods by a curette or a scalpel blade [8,34,35,36,37,38,39,40,41,42,43,44,45,46], chemical methods using ethanol [6], and electrical stimulation [47] were employed to induce endometrial injury. As compared to those studies, the present study induced more severe endometrial injury in that all the endometrial layers were circumferentially removed. Nevertheless, we here demonstrated that DES had the potential to rescue the severe endometrial damage, albeit partially.

Furthermore, decellularized scaffolds can promote multilayered 3D tissue reconstruction and have been clinically applied to the tissue engineering of several organs [48]. However, the reconstruction of the entire uterus using decellularized scaffolds is difficult and challenging. Considering the importance of the endometrium for the achievement and maintenance of pregnancy and the prevalence of endometrium-associated disorders such Asherman’s syndrome and thin endometrium, the regeneration of the endometrium rather than the entire uterus is more realistic and important [49]. The results of this study will provide a basis for the bioengineering of human endometrium and its clinical application.

This study, however, has several limitations. First, although we successfully regenerated the stromal components of the endometrium circumferentially using DES, the regenerated endometrium lacked luminal and glandular epithelium. Firstly, the direct contact between the endometrium and the silicon tube may result in failure in re-epithelialization, because silicone products are known to provoke fibrosis together with the up-regulation of fibrosis-associated molecules, including Vm [21,22]. Indeed, intense Vm signals were observed in the luminal-like structures in not all but many of the T+D or T uterus samples as shown in Figure 4D (middle and bottom panels). Secondly, additional bioactive substances may be needed for the genesis of epithelial components. Hiraoka et al. demonstrated that STAT3, a transcription factor involved in multiple biological and pathological events, and its activation by phosphorylation are important for migration and regeneration of the endometrial epithelium [26]. In this context, stimulator(s)/enhancer(s) to activate STAT3 such as LIF should be incorporated into the DES for regeneration of the endometrial epithelial components. Alternatively, epithelial (stem/progenitor) cells could be loaded onto the DES prior to the placement. The epithelial cells could contribute to the efficient regeneration of endometrial epithelium.

Second, we did not examine the potential of the regenerated endometrium to support pregnancy. The silicone tube must be placed firmly to prevent luminal adhesion. To achieve pregnancy, the tube must be subsequently removed by another surgery before embryo transfer/mating. Such repeated surgeries and pregnancy testing may be unfavorable and complicated. Silicon products have been reported to prevent adhesion reformation after hysteroscopic adhesiolysis of intrauterine adhesion [50]. We therefore used a silicon tube to prevent intrauterine adhesion in this study. If a biodegradable material with anti-adhesion activity but without any scaffold properties was available, the repeated surgery would not be necessary because the material would prevent adhesion, generate a luminal space, and thereafter spontaneously degrade and disappear. Moreover, the endometrium regenerated in this study lacked luminal epithelium, and thus embryos likely would not have attached to and migrated through the luminal epithelium to establish a successful pregnancy. However, direct placement of embryos inside the endometrium, rather than on the surface (i.e., in the uterine cavity), has been reported to result in successful pregnancy [51], suggesting that the direct interaction between embryos and luminal epithelium may not be needed for the achievement of pregnancy. Thus, in vitro fertilization combined with the transmyometrial placement of embryos inside the endometrium may enable pregnancy in our model in which luminal epithelium was absent. The glandular epithelium, however, produces many bioactive substances to interact with stroma, leading to successful pregnancy [52]. Since the glandular epithelium was also absent in our model, it remains possible that the transmyometrial placement of embryos inside the endometrium may not lead to successful pregnancy.

Third, we did not clarify the mechanism underlying endometrium regeneration. We analyzed uteri with interventions at only one time point (28 days) after surgery. In future studies, serial analyses of the regenerated endometrium at several time points (e.g., 7, 14, and 21 days) could be carried out. On the other hand, more than 28 days may be required for the epithelium to accomplish regeneration, and thus longer time periods could also be examined.

Despite these limitations, the results of this study provide insight into the bioengineering of the uterus and specific therapeutic strategies and procedures to address uterine infertility due to functional and structural defects of the endometrium.

## 4. Materials and Methods

**Figure 6:**
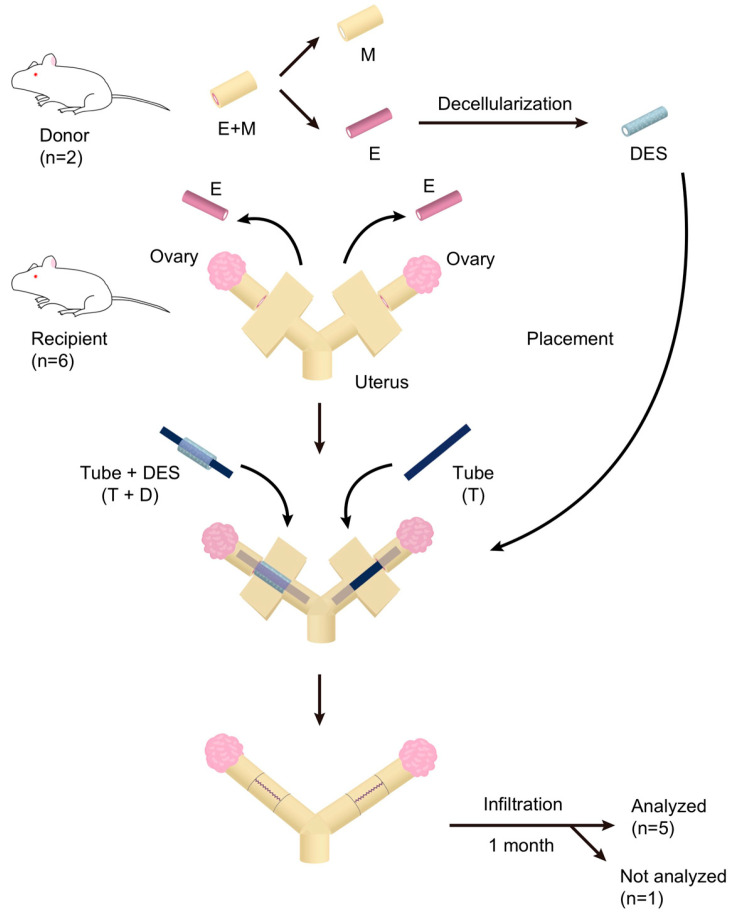
Overview of experimental design and procedures. The uterus of a donor rat was excised, and the endometrium (E) was separated from the outer layer, which consists mainly of myometrium (M), of the uterus. The separated E was decellularized and designated as a decellularized endometrium scaffold (DES). The recipient rat underwent the same separation of E but without excision of the uterus, such that a 1.5 cm long section of E alone was removed and the M of both uterine horns remained. A silicone tube or a tube-loaded DES was placed into the endometrium-deficient section of each uterine horn, and the outer muscle layer was then closed with continuous suturing. The silicone tube was used to prevent intrauterine adhesion. A month later the uterus was excised for histological and immunofluorescence microscopic analyses. The number of rats used as indicated are shown in parentheses.

An overview of the experimental design and procedures used in this study is shown in Figure 6. We prepared 1.5 cm long pieces of DES from the endometria of two donor rats. Six rats received T+D or T treatment on each uterine horn and were sacrificed one month after the treatment. One of the six recipient rats showed luminal adhesion and atrophy at the surgical site and was judged as inappropriate for further analysis. Thus, the remaining five rats, i.e., 10 uteri, were subjected to the subsequent histological and immunofluorescence analyses. In addition, four rats were used as the positive control for histological and immunofluorescence analyses.

### 4.1. Collection of Rat Uterine Endometrium

For uterine harvest, female Sprague Dawley (SD) rats (8–10 weeks-old) were obtained from Oriental Yeast (Tokyo, Japan). The rats were bred under pathogen-free conditions at the Keio University School of Medicine Animal Center. The rats were anesthetized with isoflurane and the abdominal wall was opened longitudinally along the midline. The entire endometrium was peeled off from the uterus in a tubular form (Figure 1A, left panel; Video S1). All animals were cared for in accordance with the rules and regulations defined by the Keio University School of Medicine for Animal Care. The Ethical Review Committee of the Institute approved the experimental protocols (protocol code, 12114; date of approval, 3 September 2012). The animal experiments were complied with the ARRIVE guidelines 2.0. The ARRIVE Checklist is available in Supplementary File S1.

### 4.2. Decellularization of the Uterine Endometrium

The extracted uterine endometrium was cut into 1.5 cm long cylindrical pieces that were decellularized using SDS (FUJIFILM Wako Pure Chemical Corporation, Osaka, Japan). The fragments were first immersed in PBS solution containing 0.01% SDS for 6 h at 4 °C followed by immersion in PBS with 0.1% SDS for 6 h at 4 °C, and then PBS with 1% SDS for 6 h at room temperature. All incubations were carried out with shaking at 1 Hz frequency. After the SDS treatment, the pieces were washed with DW for 15 min and 1% Triton X-100 (Sigma-Aldrich, St. Louis, MO, USA) for 30 min to remove residual SDS. The pieces were then incubated in sterile PBS solution for 15 min with shaking at 1 Hz frequency. DES was stored at 4 °C in PBS containing 1% antibiotic and antimycotic solution (Sigma-Aldrich).

### 4.3. DES Transplantation

A 1.5 cm long longitudinal incision was made in the abdomen of recipient female SD rats (8–10 weeks-old) under isoflurane anesthesia. The incision passed through the serosa and myometrium layer on the side opposite the mesentery to expose the endometrial layer. The uterine endometrium was peeled off similarly, but less extensively to that for the donor. We inserted an autoclaved silicone tube having a 0.5 mm inner diameter and 1 mm outer diameter (Sansyo, Tokyo, Japan) into the lumen of a 1.5 cm long section of DES (Figure 3A). The tube was used to distend the lumen and prevent luminal adhesions. The DES loaded with the silicone tube was placed and fixed into the endometrium-denuded site with suturing and ligation using #6-0 Prolene (Ethicon, Brussels, Belgium) (T+D group) (Figure S1B). As a negative control, an autoclaved silicone tube alone was placed and fixed into the endometrium-denuded site with suturing and ligation using #6-0 Prolene (Figure S1A).

After transplantation of the tube alone (T) or tube-loaded DES (T+D), the muscle layer and serosa were continuously sutured with #6-0 Prolene to cover the grafted area. The rats were sacrificed one month after transplantation and the uteri were removed for histological and immunofluorescent analyses.

### 4.4. Histological and Immunofluorescent Analyses

To remove the uteri, cuts were made at the ovarian and vaginal ligature points and the grafted sites were extracted. Each site was embedded in Tissue-Tek OCT compound (Sakura Finetech, Torrance, CA, USA). The embedded tissue samples were frozen and serially sectioned into 6 µm thick slices every 1 mm with a Leica cryostat (Leica Microsystems, Wetzlar, Germany). The cryosections were washed in PBS twice for 3 min, fixed in 4% paraformaldehyde for 10 min at room temperature, and washed with PBS twice. For histological analysis using H&E staining, the slides were stained with Mayer’s hematoxylin solution (FUJIFILM Wako) for 10 min, washed under running tap water for 30 min and then stained with 0.5% Eosin Y, ethanol solution (FUJIFILM Wako) for 5 min. The slides were shuffled up and down 10 times in 70%, 80%, and 90% ethanol solution, and then rinsed three times for 1 min each with 100% ethanol followed by three 1 min rinses with 100% xylene. The samples were mounted with Mount-Quick (Cosmo Bio, Tokyo, Japan).

For immunofluorescence analyses, the fixed slides were permeabilized with 0.2% Triton X-100 in PBS for 10 min. After washing with PBS twice, the samples were blocked with 1% BSA/PBS for 30 min, and then incubated overnight at 4 °C with pre-titrated primary antibodies diluted 1/300. The primary antibodies included mouse monoclonal anti-cytokeratin (Dako, Santa Clara, CA, USA, MNF116), mouse monoclonal anti-vimentin (Dako, V9), mouse monoclonal anti-smooth muscle actin (Dako), rabbit polyclonal anti-collagen type I (Abcam, Cambridge, UK), and rabbit polyclonal anti-laminin (Sigma-Aldrich). After washing, the primary antibodies were visualized by incubation for 60 min with secondary antibodies diluted 1/1000, including anti-mouse IgG Alexa Fluor 488 conjugate (Invitrogen Carlsbad, CA, USA) or anti-rabbit IgG Alexa Fluor 546 conjugate (Invitrogen). Mouse vimentin-Cy3 antibody diluted 1/400 was also used as a secondary antibody. After washing with PBS, the slides were stained with Hoechst 33342 (Ho) (Thermo Fisher Scientific, Waltham, MA, USA) for 5 min, washed with PBS twice, and mounted with fluorescence mounting medium (Dako). Images were acquired using a fluorescence microscope BZ-X810 (Keyence, Osaka, Japan) and BZ-X800 analyzer application (Keyence).

The boundary between the myometrium and endometrium in each section was determined by two blinded examiners (S.K. and S.T.) in addition to the observer who collected the images. SMA-positive areas were considered to represent the myometrium layer. SMA-negative and Vm-positive areas were considered to represent the endometrial stromal area. The size of each area and the number of nuclei were analyzed using ImageJ (National Institutes of Health, Bethesda, Maryland, USA). The relative area ratio and the relative nuclear number ratio were calculated by dividing the corresponding area and nuclei number for each uterine horn, respectively.

### 4.5. Statistics

Values are expressed as mean ± SEM together and displayed in dot plots of individual values obtained from independent experiments. Statistical analysis was carried out using Statistical Package for the Social Sciences (SPSS) ver. 27 (SPSS Inc., Chicago, IL, USA). For statistical analysis, the Levene’s test and Kolmogorov–Smirnov test were performed, followed by parametric Student’s *t*-test or nonparametric Mann–Whitney test for comparison between the two groups. *p* values of < 0.05 were considered statistically significant.

## 5. Conclusions

In this study, we investigated whether the placement of DES into a circumferential defect of the endometrium in rats can promote circumferential regeneration and recovery of the defect in the endometrium. We found that DES could enhance the circumferential regeneration of the endometrial stroma, but had little or no ability to induce epithelization within one month of transplantation. Furthermore, prevention of adhesions allowed the endometrial stroma to regenerate circumferentially even in the absence of the DES, although to a lesser degree than that seen in the presence of DES. The use of DES together with strategies to prevent adhesions may be beneficial for the regeneration of the endometrium in a uterus that largely lacks endometrium.

## Data Availability

Not applicable.

## Supplementary Materials

The following supporting information can be downloaded at: https://www.mdpi.com/article/10.3390/ijms24087605/s1, Figure S1: Suturing method for placement and fixation of a silicone tube alone or tube-loaded DES in the endometrium-deficient area of the uterine horn; Video S1: Procedure for endometrium separation. Supplementary File S1: The ARRIVE Checklist.
