# Supplementary material for: A Decellularized Uterine Endometrial Scaffold Enhances Regeneration of the Endometrium in Rats
Source: Int J Mol Sci. 2023 Apr 20;24(8):7605. doi: 10.3390/ijms24087605 (PMC10145056; doi:10.3390/ijms24087605)
Supplement: Supplementary file 1 [file ijms-24-07605-s001.zip › ijms-2179462-Supplementary Materials figure and video description.pdf]

International Journal of
Molecular Sciences

Supplementary Materials figure and video description

Figure S1: Suturing method for placement and fixation of a silicone tube alone or tube-loaded DES in the endometrium-deficient area of the uterine horn; Video S1: Procedure for endometrium separation.
