# Supplementary figures and images for: A Decellularized Uterine Endometrial Scaffold Enhances Regeneration of the Endometrium in Rats
Source: Int J Mol Sci. 2023 Apr 20;24(8):7605. doi: 10.3390/ijms24087605 (PMC10145056; doi:10.3390/ijms24087605)

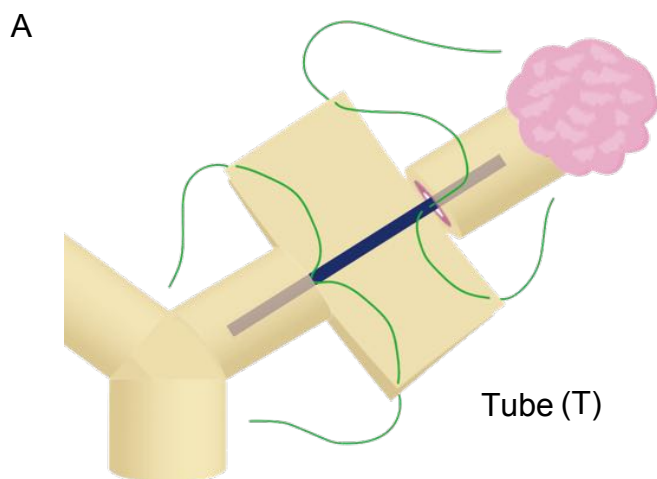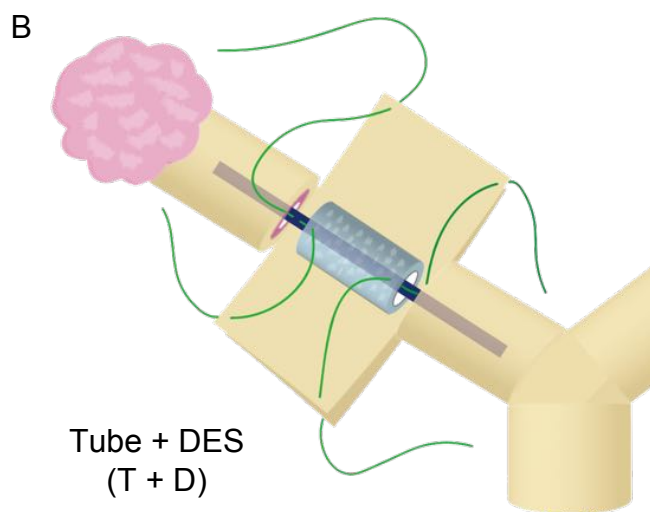

Figure S1. Yoshimasa et al.

Supplement: Supplementary file 1 [file ijms-24-07605-s001.zip › ijms-2179462-supplementary figure.pdf]
